# Retraction: Identification of sumoylation sites in CCDC6, the first identified RET partner gene in papillary thyroid carcinoma, uncovers a mode of regulating CCDC6 function on CREB1 transcriptional activity

**DOI:** 10.1371/journal.pone.0357529

**Published:** 2026-09-03

**Authors:** The PLOS One Editors

Following the publication of this article [1] concerns were raised about results presented in Figs 1, 3, 4, and S3. Specifically,

- In Fig 1C, the following bands appear more similar than would be expected from independent results:
  - Ni-NTA pull down IB anti-HIS lanes 2 and 4.
  - Total extract IB anti-CCDC6 lanes 2 and 3.
- The following panels appear more similar than would be expected from independent results:
  - Fig 3C CCDC6wt Flag and Fig 3C CCDC6 TM Flag when levels are adjusted to visualize background.
  - Fig 3C CCDC6 TM GFP and Hoechst, and Fig S3 CCDC6wt GFP and Hoechst respectively.
- Fig 4B CREB1 lanes 2-5 and Fig 4C CREB1 lanes 1-4 appear similar despite representing different experimental results.

The corresponding author confirmed that the Fig S3 CCDC6wt GFP and Hoechst panels were incorrect as a result of a figure preparation error and provided replacement panels.

Regarding the concerns with Figs 4B and 4C, the corresponding author stated that the CREB1 panels serve as a shared loading control for these figures derived from the same master lysate mix run on the same gel. They clarified that the Fig 4C CREB1 panel was spliced, such that the first lane of the Fig 4C CREB1 panel corresponds to the first lane of the Fig 4B CREB1 panel. They stated that the uncropped blots underlying the Figs 4B and 4C results are no longer available.

The corresponding author’s response to the concerns with Fig 1C and Fig 3C did not resolve the editorial concerns.

In light of the above unresolved concerns, the *PLOS One* editors retract this article.

AC did not agree with the retraction. CL, FM, VL, SP, DS, and AF either did not respond directly or could not be reached.
